# NRG Oncology Survey of Monte Carlo Dose Calculation Use in US Proton Therapy Centers

**DOI:** 10.14338/IJPT-D-21-00004

**Published:** 2021-05-25

**Authors:** Liyong Lin, Paige A. Taylor, Jiajian Shen, Jatinder Saini, Minglei Kang, Charles B. Simone, Jeffrey D. Bradley, Zuofeng Li, Ying Xiao

**Affiliations:** 1 Emory University, Atlanta, GA, USA; 2 MD Anderson Cancer Center, Houston, TX, USA; 3 Mayo Clinic Arizona, Phoenix, AZ, USA; 4 Seattle Cancer Care Alliance Proton Therapy Center, Seattle, WA, USA; 5 New York Proton Center, New York, NY, USA; 6 Department of Radiation Oncology, University of Florida College of Medicine, Gainesville, FL, USA; 7 University of Pennsylvania, Philadelphia, PA, USA

**Keywords:** Monte Carlo, proton therapy, DECT, MAR, surgical implant

## Abstract

**Purpose/Objective(s):**

Monte Carlo (MC) dose calculation has appeared in primary commercial treatment-planning systems and various in-house platforms. Dual-energy computed tomography (DECT) and metal artifact reduction (MAR) techniques complement MC capabilities. However, no publications have yet reported how proton therapy centers implement these new technologies, and a national survey is required to determine the feasibility of including MC and companion techniques in cooperative group clinical trials.

**Materials/Methods:**

A 9-question survey was designed to query key clinical parameters: scope of MC utilization, validation methods for heterogeneities, clinical site-specific imaging guidance, proton range uncertainties, and how implants are handled. A national survey was distributed to all 29 operational US proton therapy centers on 13 May 2019.

**Results:**

We received responses from 25 centers (86% participation). Commercial MC was most commonly used for primary plan optimization (16 centers) or primary dose evaluation (18 centers), while in-house MC was used more frequently for secondary dose evaluation (7 centers). Based on the survey, MC was used infrequently for gastrointestinal, genitourinary, gynecology and extremity compared with other more heterogeneous disease sites (*P* < .007). Although many centers had published DECT research, only 3/25 centers had implemented DECT clinically, either in the treatment-planning system or to override implant materials. Most centers (64%) treated patients with metal implants on a case-by-case basis, with a variety of methods reported. Twenty-four centers (96%) used MAR images and overrode the surrounding tissue artifacts; however, there was no consensus on how to determine metal dimension, materials density, or stopping powers.

**Conclusion:**

The use of MC for primary dose calculation and optimization was prevalent and, therefore, likely feasible for clinical trials. There was consensus to use MAR and override tissues surrounding metals but no consensus about how to use DECT and MAR for human tissues and implants. Development and standardization of these advanced technologies are strongly encouraged for vendors and clinical physicists.

## Introduction

The number of proton therapy centers has increased rapidly in recent years. The proton pencil beam scanning technique has been widely implemented in almost all new proton centers, and it is expected to be the future trend in proton therapy [1]. The dose calculation algorithm plays an important role in the accuracy and quality of proton beam therapy. The analytic dose engine, such as pencil beam superposition convolution (PSC), has the advantage of fast calculation speed and is widely implemented in commercial treatment-planning systems (TPSs), such as Eclipse (Varian Medical Systems, Palo Alto, California), RayStation (RaySearch Laboratories, Stockholm, Sweden), and Pinnacle (Philips, Amsterdam, Netherlands). However, Taylor et al [2] reported that proton centers using the analytical dose engine had low passing rates on the Imaging and Radiation Oncology Core (IROC) lung phantom. On the contrary, Monte Carlo (MC) dose calculation has been found to improve dose calculation accuracy in low-density heterogeneities, and correspondingly, has a favorable IROC lung phantom pass rate. Hence, the National Cancer Institute recommends using MC in its sponsored lung-related clinical trials. Similarly, MC has been reported to be advantageous in highly heterogeneous patients and is recommended for patients with a large metal implant [3–7].

Since 2018, MC for pencil beam scanning techniques has been implemented by major commercial TPSs. The Eclipse TPS currently offers MC for final dose calculation, but plan optimization is still based on analytical methods. The RaySearch TPS offers MC for both plan optimization and final dose calculation; however, if quick optimization is desired, analytical PSC is an option for plan optimization. Commissioning TPS MCs has been reported to improve dosimetric accuracies over PSC [8–10]. Besides the aforementioned commercial MCs, in-house MCs, such as TOPAS [11] (TOPAS MC Inc, Boston, Massachusetts), MCsquare [4] (Université Catholique de Louvain, Belgium), and others, from early in-house development at the Paul Scherrer Institute [5] to general-purpose MC (Geant4/Gate [12] and Geant4/FLUKA [13]) to graphics processing unit (GPU)–based MC (gPMC at Massachusetts General Hospital in Boston [14] and the in-house MC at the Mayo Clinic, Phoenix, Arizona [15]) have been widely used for clinical dose calculations.

The advantages of MC over PSC have motivated clinical trial sponsors to consider requiring MC in all future proton therapy clinical trials. In 2018, the NRG Oncology Medical Physics Subcommittee established a working group to examine the feasibility of implementing MC in all clinical trials involving proton therapy. The working group comprised radiation oncologists and a therapeutic medical physicist with expertise in proton therapy. The working group designed a survey for National Clinical Trial Network (NCTN) members to query the current clinical practice and distributed it to proton therapy centers in the United States. The goal of this survey was to inform clinical site-specific practice guidelines for NRG Oncology–sponsored clinical trials.

MC is intrinsically different from PSC and requires specific considerations for implementation. For example, unlike the conversion of computed tomography (CT) Hounsfield units (HUs) to stopping power in the analytical solution, the MC method looks for elemental composition and mass density to determine stopping power and scattering cross-sections. Heterogeneity is typically placed into 2 categories: human tissues, described by stoichiometric calibration in single-energy CT [16], and artificial implants, which can have artifacts over surrounding tissues and HUs that cannot be represented by that of human tissues. Guidelines are desired for both users and vendors regarding the definition of artificial implants, including geometry and elemental composition.

Recent developments in dual-energy CT (DECT) [17–21] and CT metal artifact reconstruction (MAR) [22–24] show potential to improve range uncertainty and enable treatment of patients with large implants [25]. Guidelines are needed on the use of tissue-mimicking phantoms for the validation of proton therapy dose calculation and on how to properly use DECT and MAR images to achieve the most accurate patient dose distribution leading to best patient outcome and safety. Hence, the last purpose of this survey was to find out how these complementary imaging techniques were used along with the practice of MC.

## Methods and Materials

Given the novelty and importance of MC to clinical trials, NRG Oncology has formed a work group to develop a practice guideline on the application of MC to national cooperative group clinical trials. The overall goal was to improve treatment dose calculation accuracies for patients without artificial materials and to safely treat patients with artificial materials that were previously reported to be associated with inferior local control when delivering proton therapy alone [26] and recommended to receive mixed photon/proton therapies to mitigate proton dose inaccuracies [27, 28].

As no publications to date have reported how proton therapy centers implement MC and complementary imaging technologies such as DECT and MAR, a current practice pattern assessment is required to determine the feasibility of including them in clinical trials. To address the issue, a survey was designed to query key clinical parameters: scope of MC utilization, validation methods in homogeneous and heterogeneous phantoms, clinical site-specific imaging guidance, proton range uncertainties, and how metal implants are handled.

The survey data provide insightful information on several questions. First, how widely available MC is and whether it has been implemented in current proton therapy centers. Second, what type of cancers are being optimized and calculated by MC. This information informs whether the field is ready for implementing MC for clinical trials and what disease sites may show clinical advantages with MC.

The full version of the survey questions is included in the **Supplemental Appendix**. Questions 1, 2, and 4 surveyed the type and use of MC. Questions 3 and 7 surveyed the need for MC and values of range uncertainty for specific disease sites. Questions 5 and 8 covered the commissioning and validation of MC, including heterogeneous validation and patient quality assurance methods. Question 6 surveyed site-specific imaging methods (eg, DECT, MAR, magnetic resonance imaging, others). Question 9 covered different issues encountered treating patients with implants: material, composition, dimension, range uncertainties, and mitigation strategies. Pull-down lists of values and frequency (always, often, sometimes, never) were used along with free-form text boxes to allow some users to input unique answers or clarify the answers given.

The survey was distributed to all 29 operational US proton therapy centers on 13 May 2019 following European and NRG Oncology precedents [29–31]. The survey was modified slightly for clarification and redistributed on 18 October 2019. The survey was distributed by IROC, which monitors proton therapy centers that participate in NCTN protocols.

Two-sample *t*-tests were performed by using MATLAB and Statistics Toolbox Release 2018b (The MathWorks, Inc, Natick, Massachusetts). A *P* value <.05 was considered statistically significant.

## Results

### MC Implementation and Availability in Proton Centers

**Figure 1.**
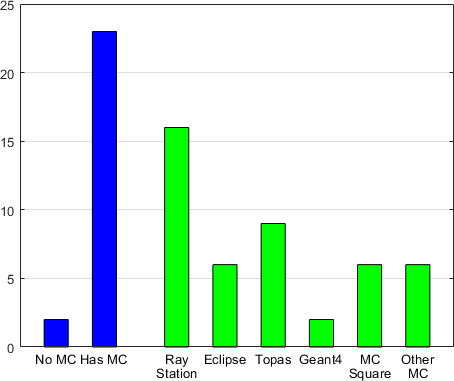
Monte Carlo implementation and availability in 25 proton centers.

The overall MC availability in proton centers and the specific implementation of commercial and in-house systems are shown in **Figure 1**. In total, 25 centers responded (86% participation rate). Of those, 23 centers reported (92%) having at least 1 MC system. Of the centers, 12 had 1 MC system, 3 had 2, 5 had 3 and 4 had 5 MC systems. Only 2 centers did not report use of MC. The commercial MC systems RayStation and Eclipse were used in 16 and 6 centers, respectively. Most of the in-house MC systems were freely available softwares: Topas (n = 9), Geant4 (n = 2), and MCsquare (n = 6). The other 6 in-house MC systems reported were 4 GPU-based MCs [14, 15] and 2 developmental MCs.

### General Purpose and Use of MC in Proton Centers

**Figure 2.**
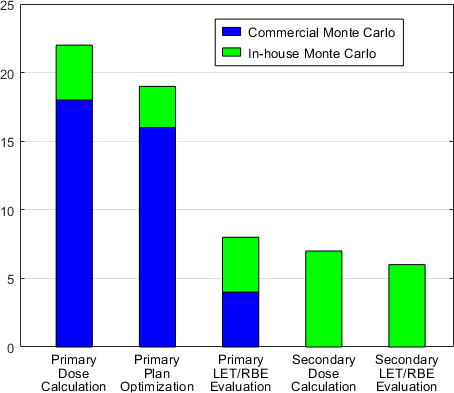
General purpose and usage of Monte Carlo in 23 proton centers.

**Figure 2** shows the general usage of MC systems in proton centers. Primary dose evaluation is mostly done by commercial MC systems rather than in-house MC systems (18 vs 4 centers). Specifically, RayStaton was used by 15 centers, Eclipse was used by 3 centers, and the in-house MC systems used by 4 centers were Topas/MCsquare, GPU-based MC [14, 15], and development MC, respectively. Of note: Eclipse MC systems currently do not support plan optimization. MC plan optimization was performed in 16 centers using RayStation and in 3 centers using in-house MC (2 MCsquare and 1 GPU-based MC [15]). Linear energy transfer (LET) relative biological effectiveness (RBE) evaluation was done in different forms: half of the 8 centers used RayStation and the others used in-house MC for primary evaluation; 6 centers used in-house MC systems as secondary LET/RBE evaluation.

### Disease-Site Specific Use of MC in Proton Centers

**Figure 3.**
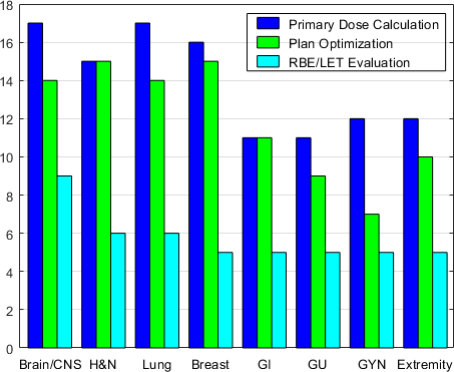
Disease site specific usage of Monte Carlo in 23 proton centers.

**Figure 3** shows the use of MC in specific disease sites. Significantly more centers (*P* = .007) used MC for both dose calculation and plan optimization when treating brain/central nervous system (CNS), head and neck, lung, and breast cancers than for gastrointestinal, genitourinary (GU), gynecologic (GYN), and extremity cancers. This is potentially due to the latter group of disease sites being of more homogeneous tissue density than the first group. Primary LET/RBE evaluation is used more for brain/CNS tumors than for all the other disease sites (*P* < .001), potentially due to risks to serial organs, such as optical structures, brain stem and spinal cord.

### MC for Modeling Accessories

MC was always or sometimes used to model range shifters in 60% and 20% of proton centers, respectively. Apertures were used in 16 centers, and MC was used to model apertures at most of these centers (69%). Two proton centers also used MC for patient-specific bolus and adaptive aperture (eg, multi-leaf collimators).

### Commissioning and Validation Methods for Heterogeneities

Most centers used electron density phantoms for stoichiometric calibration (84%). IROC phantoms were widely used for heterogeneity validation (92%). Ten centers (40%) and 7 centers (28%) used real animal tissues for stoichiometric calibration and heterogeneity validation, respectively. Other than IROC and electron density phantoms, there was no consensus over the use of heterogeneous phantoms.

### Imaging Method to Reduce Uncertainty from Heterogeneities

**Figure 4.**
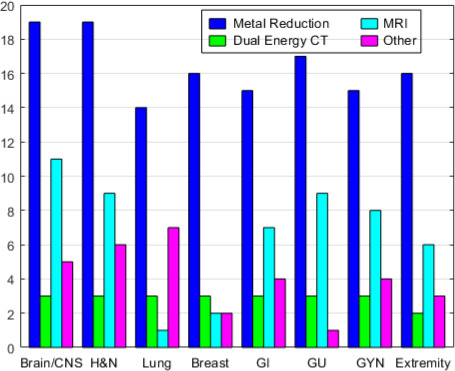
Imaging method to reduce the uncertainty from heterogeneities in 25 proton centers.

**Figure 4** shows the number of proton centers that used various image methods to reduce uncertainties from heterogeneity for brain/CNS, head and neck, lung, breast, gastrointestinal, GU, GYN, and extremity cancers. Metal artifact reduction (eg, iterative metal artifact reduction and orthopedic metal artifact reduction) is the imaging technique most proton centers used to reduce the artifacts (76%). Dual-energy CT (DECT) was rarely implemented for clinical use (3/25). Magnetic resonance imaging was used by <44% of centers to reduce uncertainty from heterogeneities. Seven centers (<28%) used positron emission tomography/CT, proton radiography, and prompt Gamma to reduce uncertainty from tissue heterogeneity.

### Range Uncertainty in Treatment Planning

Most centers (92%) used range uncertainties of 3% or 3.5% for treating all disease sites except lung. For lung cancer, 7 centers (28%) used a larger range uncertainty of 4% or 5%.

### Criteria for Patient-Specific Quality Assurance

For plans that did not pass the robustness evaluation, most centers (96%) required a replan before patient-specific quality assurance (QA). Most centers (84%) used >90% to 95% Gamma passing rate with 3-mm distance to agreement and 3% dose accuracy as patient-specific QA criteria. If the patient QA was outside criteria, the most common action (taken by 84% of centers) was to renormalize the plan monitor unit according to the patient QA results. The second most common action if the patient QA was outside criteria was to redo the treatment plan (68% of centers). Many centers (40%) also reported that they would hold patient treatment if the patient QA was outside 3%/3 mm.

### Procedure to Handle Metal Implants

Most centers (96%) did not have an absolute maximum size of metal implants to determine the feasibility of treatment and instead evaluated case by case. When proton beams had to pass through metal implants, 71% of centers used a larger range uncertainty margin to take into account the larger range uncertainty. All proton centers (100%) considered using more beams from different passing angles to smooth out the dose perturbations caused by metal implants. Most centers (81%) used MC methods to improve dose accuracy with metal implants in the beamline. All proton centers except 1 (96%) would override the artifacts in the tissue surrounding the metal implants. However, for the metal implant itself, there was no consensus on precisely determining metal dimension or assigning materials density and stopping powers.

## Discussion

A majority of proton therapy centers were using MC (commercial or in-house) for various purposes in their clinics. MC was used for treatment planning, plan optimization, LET/RBE evaluation, and secondary dose calculations. The survey responses showed that MC use for some disease sites, such as GU and GYN, was lower than for other disease sites. While some proton centers were not treating GYN cancers with proton therapy (currently there are no NCTN clinical trials for GYN cancer that allow proton therapy), GU diseases, such as prostate cancer, were currently being studied in multiple active phase III trials [32, 33]. Perhaps one reason for less use of MC in these disease sites is the relative tissue homogeneity of the anatomy compared with more heterogeneous disease sites, such as lung tumors. MC was also dominantly used in brain/CNS, head and neck, and breast cancers, probably due to spine implants, dental implants, and breast implants, respectively. However, from a resource perspective, it is cumbersome to maintain one TPS for some disease sites and a second for others. As many centers have already implemented MC for clinical use, it would be ideal for clinical trial data collection and consistency for all proton centers to use the same treatment-planning algorithm. Given this, we encourage proton centers to consider using MC for all disease sites. However, this may slow down the clinic because MC dose engines are more time consuming in general than the analytic ones.

One of the important issues identified in this survey was lack of consensus regarding the use of DECT and consensus of using MAR for artificial materials. Despite the promise of DECT, it was surprising that so few proton centers had implemented this clinically. We speculate that this is partly due to a lack of adequate support for DECT in commercial TPSs. Furthermore, current DECT uses 2-parameter [34] to extract material composition, which is adequate for HU accuracy of human tissues but might not be sufficient for artificial tissues. For patients with large and high Z artificial materials, MC is desired.

However, current DECT techniques cannot provide the mass density and material composition that is needed for MC. Furthermore, a pixilated proton counting detector has been introduced to validate derived material information for anthropomorphic phantoms [35]. DECT and TPS vendors are encouraged to provide a solution to extract and implement material information for human and artificial tissues so that MC can be properly used in clinics. Proton centers with DECT capabilities are encouraged to evaluate DECT for MC-based treatment planning purposes.

The survey had some design limitations. For example, the survey combined the imaging techniques of positron emission tomography/CT, proton radiography, and prompt gamma into a single option when querying methods used to minimize patient-specific heterogeneity uncertainty. Therefore, it is impossible to distinguish which method is available at individual centers. Another limitation was with the question regarding patient-specific QA. The survey used the broad term “patient specific QA,” which can be interpreted in many ways, for example, the comparison between the dose calculated by TPSs with the measurements by detectors, the dose calculated by the second monitor unit check, or the dose recalculated by the log files.

## Conclusion

Based on the survey results, the use of MC for primary dose calculation and optimization was available and feasible for patients without artificial materials in most proton therapy centers. For comparability, consistency, and accuracy in clinical trials, we encourage proton centers to commission or adopt MC for clinical use. For patients with metal implants, there is consensus to use metal artifact reduction and to override tissues surrounding metals. However, there is no consensus about DECT regarding the use of virtual monoenergetic images or the extraction of material information for artificial and human tissues. Development and standardization of these advanced technologies are strongly encouraged for vendors and clinical physicists alike.

## References

[1] Farr JB, Flanz JB, Gerbershagen A, Moyers MF (2018). New horizons in particle therapy systems. *Med Phys*.

[2] Taylor PA, Kry SF, Followill DS (2017). Pencil beam algorithms are unsuitable for proton dose calculations in lung. *Int J Radiat Oncol Biol Phys*.

[3] Schuemann J, Giantsoudi D, Grassberger C, Moteabbed M, Min CH, Paganetti H (2015). Assessing the clinical impact of approximations in analytical dose calculations for proton therapy. *Int J Radiat Oncol Biol Phys*.

[4] Huang S, Souris K, Li S, Kang M, Barragan Montero AM, Janssens G, Lin A, Garver E, Ainsley C, Taylor P, Xiao Y, Lin L (2018). Validation and application of a fast Monte Carlo algorithm for assessing the clinical impact of approximations in analytical dose calculations for pencil beam scanning proton therapy. *Med Phys*.

[5] Tourovsky A, Lomax AJ, Schneider U, Pedroni E (2005). Monte Carlo dose calculations for spot scanned proton therapy. *Phys Med Biol*.

[6] Newhauser WD, Giebeler A, Langen KM, Mirkovic D, Mohan R (2008). Can megavoltage computed tomography reduce proton range uncertainties in treatment plans for patients with large metal implants?. *Phys Med Biol*.

[7] Righetto R, Clemens LP, Lorentini S, Fracchiolla F, Algranati C, Tommasino F, Dionisi F, Cianchetti M, Schwarz M, Farace P (2020). Accurate proton treatment planning for pencil beam crossing titanium fixation implants. *Phys Med*.

[8] Saini J, Maes D, Egan A, Bowen SR, St James S, Janson M, Wong T, Bloch C (2017). Dosimetric evaluation of a commercial proton spot scanning Monte-Carlo dose algorithm: comparisons against measurements and simulations. *Phys Med Biol*.

[9] Yepes P, Adair A, Grosshans D, Mirkovic D, Poenisch F, Titt U, Wang Q, Mohan R (2018). Comparison of Monte Carlo and analytical dose computations for intensity modulated proton therapy. *Phys Med Biol*.

[10] Chang C-W, Huang S, Harms J, Zhou J, Zhang R, Dhabaan A, Slopsema R, Kang M, Liu T, McDonald M, Langen K, Lin L (2020). A standardized commissioning framework of Monte Carlo dose calculation algorithms for proton pencil beam scanning treatment planning systems. *Med Phys*.

[11] Perl J, Shin J, Schümann J, Faddegon B, Paganetti H (2012). TOPAS: an innovative proton Monte Carlo platform for research and clinical applications. *Med Phys*.

[12] Grevillot L, Boersma DJ, Fuchs H, Aitkenhead A, Elia A, Bolsa M, Winterhalter C, Vidal M, Jan S, Pietrzyk U, Maigne L, Sarrut D (2020). Technical note: GATE-RTion: a GATE/Geant4 release for clinical applications in scanned ion beam therapy. *Med Phys*.

[13] Parodi K, Mairani A, Brons S, Hasch BG, Sommerer F, Naumann J, Jäkel O, Haberer T, Debus J (2012). Monte Carlo simulations to support start-up and treatment planning of scanned proton and carbon ion therapy at a synchrotron-based facility. *Phys Med Biol*.

[14] Jia X, Schümann J, Paganetti H, Jiang SB (2012). GPU-based fast Monte Carlo dose calculation for proton therapy. *Phys Med Biol*.

[15] Beltran C, Tseung HWC, Augustine KE, Bues M, Mundy DW, Walsh TJ, Herman MG, Laack NN (2016). Clinical implementation of a proton dose verification system utilizing a GPU accelerated Monte Carlo engine. *Int J Part Ther*.

[16] Schneider U, Pedroni E, Lomax A (1996). The calibration of CT Hounsfield units for radiotherapy treatment planning. *Phys Med Biol*.

[17] Yang M, Virshup G, Clayton J, Zhu XR, Mohan R, Dong L (2010). Theoretical variance analysis of single- and dual-energy computed tomography methods for calculating proton stopping power ratios of biological tissues. *Phys Med Biol*.

[18] Yang M, Virshup G, Clayton J, Zhu XR, Mohan R, Dong L (2011). Does kVMV dual-energy computed tomography have an advantage in determining proton stopping power ratios in patients?. *Phys Med Biol*.

[19] Yang M, Zhu XR, Park PC, Titt U, Mohan R, Virshup G, Clayton JE, Dong L (2012). Comprehensive analysis of proton range uncertainties related to patient stopping-power-ratio estimation using the stoichiometric calibration. *Phys Med Biol*.

[20] Shen C, Li B, Chen L, Yang M, Lou Y, Jia X (2018). Material elemental decomposition in dual and multi-energy CT via a sparsity dictionary approach for proton stopping power ratio calculation. *Med Phys*.

[21] Xie Y, Ainsley C, Yin L, Zou W, McDonough J, Solberg TD, Lin A, Teo BK (2018). Ex vivo validation of a stoichiometric dual energy CT proton stopping power ratio calibration. *Phys Med Biol*.

[22] Nielsen JS, Van Leemput K, Edmund JM (2020). MR-based CT metal artifact reduction for head-and-neck photon, electron, and proton radiotherapy. *Med Phys*.

[23] Gjesteby L, Shan H, Yang Q, Xi Y, Jin Y, Giantsoudi D, Paganetti H, De Man B, Wang G (2019). A dual-stream deep convolutional network for reducing metal streak artifacts in CT images. *Phys Med Biol*.

[24] Andersson KM, Dahlgren CV, Reizenstein J, Cao Y, Ahnesjö A, Thunberg P (2018). Evaluation of two commercial CT metal artifact reduction algorithms for use in proton radiotherapy treatment planning in the head and neck area. *Med Phys*.

[25] Dietlicher I, Casiraghi M, Ares C, Bolsi A, Weber DC, Lomax AJ, Albertini F (2014). The effect of surgical titanium rods on proton therapy delivered for cervical bone tumors: experimental validation using an anthropomorphic phantom. *Phys Med Biol*.

[26] Snider JW, Schneider RA, Poelma-Tap D, Stieb S, Murray FR, Placidi L, Albertini F, Lomax A, Bolsi A, Kliebsch U, Malyapa R, Weber DC (2018). Long-term outcomes and prognostic factors after pencil-beam scanning proton radiation therapy for spinal chordomas: a large, single-institution cohort. *Int J Radiat Oncol Biol Phys*.

[27] DeLaney TF, Liebsch NJ, Pedlow FX, Adams J, Weyman EA, Yeap BY, Depauw N, Nielsen GP, Harmon DC, Yoon SS, Chen Y-L, Schwab JH, Hornicek FJ (2014). Long-term results of Phase II study of high dose photon/proton radiotherapy in the management of spine chordomas, chondrosarcomas, and other sarcomas. *J Surg Oncol*.

[28] Indelicato DJ, Rotondo RL, Begosh-Mayne D, Scarborough MT, Gibbs CP, Morris CG, Mendenhall WM (2016). A prospective outcomes study of proton therapy for chordomas and chondrosarcomas of the spine. *Int J Radiat Oncol Biol Phys*.

[29] Bolsi A, Peroni M, Amelio D, Dasu A, Stock M, Toma-Dasu I, Nyström PW, Hoffmann A (2018). Practice patterns of image guided particle therapy in Europe: a 2016 survey of the European Particle Therapy Network (EPTN). *Radiother Oncol*.

[30] Monroe JI, Boparai K, Xiao Y, Followill D, Galvin JM, Klein EE, Low DA, Moran JM, Zhong H, Sohn JW (2018). NRG oncology medical physicists' manpower survey quantifying support demands for multi-institutional clinical trials. *Pract Radiat Oncol*.

[31] Yock AD, Mohan R, Flampouri S, Bosch W, Taylor PA, Gladstone D, Kim S, Sohn J, Wallace R, Xiao Y, Buchsbaum J (2019). Robustness analysis for external beam radiation therapy treatment plans: describing uncertainty scenarios and reporting their dosimetric consequences. *Pract Radiat Oncol*.

[32] (2021). A prospective comparative study of outcomes with proton and photon radiation in prostate cancer (COMPPARE). ClinicalTrials.gov.identifier.

[33] (2020). Proton therapy vs. IMRT for low or intermediate risk prostate cancer (PARTIQoL). ClinicalTrials.gov.identifier.

[34] Hünemohr N, Krauss B, Tremmel C, Ackermann B, Jäkel O, Greilich S (2014). Experimental verification of ion stopping power prediction from dual energy CT data in tissue surrogates. *Phys Med Biol*.

[35] Charyyev S, Chang CW, Harms JM, Oancea C, Yoon ST, Yang X, Zhang T, Zhou J, Lin L (2021). A novel proton counting detector and method for the validation of tissue and implant material maps for Monte Carlo dose calculation. *Phys Med Biol*.

