# Supplementary material for: NRG Oncology Survey of Monte Carlo Dose Calculation Use in US Proton Therapy Centers
Source: Int J Part Ther. 2021 May 25;8(2):73–81. doi: 10.14338/IJPT-D-21-00004 (PMC8489489; doi:10.14338/IJPT-D-21-00004)
Supplement: Supplementary file 1 [file ijpt-08-02-09_s01.docx]

Appendix

| Question | Available Answers |
| --- | --- |
| 1. What Monte Carlo systems are available for clinical plan optimization or evaluation? | Commercial: Eclipse, RayStation, None  In-house: Topas, Geant4, MCsquare, None.  Other |
| 2. Is Monte Carlo used for the following treatment planning purposes?  Primary plan optimization  Primary dose evaluation  Primary RBE/LET optimization  Secondary dose evaluation  Secondary plan optimization  Secondary RBE/LET optimization | Always  Sometimes  Never |
| 3. Is Monte Carlo ever used in any (above listed) treatment planning process for the following disease sites?  Brain/CNS  Head & Neck  Lung Lymphoma  Breast  GI  GU  GYN  Extremities | Yes  No |
| 4. Is Monte Carlo used to model the following nozzle accessories?  Aperture  Range Shifter  Other purpose | Always  Sometimes  Never  NA |
| 5. What methods were used for commissioning/validation of heterogeneities?  Stoichiometric calibration: Electron Density Phantoms, Rando Phantoms, IROC Phantoms, Real Animal Tissues, Other customized phantoms  Film inside phantom: Electron Density Phantoms, Rando Phantoms, IROC Phantoms, Real Animal Tissues, Other customized phantoms  TLD/OSLD inside phantom: Electron Density Phantoms, Rando Phantoms, IROC Phantoms, Real Animal Tissues, Other customized phantoms  1D ionization chamber outside phantom: Electron Density Phantoms, Rando Phantoms, IROC Phantoms, Real Animal Tissues, Other customized phantoms  Scintillation array outside phantom: Electron Density Phantoms, Rando Phantoms, IROC Phantoms, Real Animal Tissues, Other customized phantoms  Ionization chamber array outside phantom: Electron Density Phantoms, Rando Phantoms, IROC Phantoms, Real Animal Tissues, Other customized phantoms  Film outside phantom: Electron Density Phantoms, Rando Phantoms, IROC Phantoms, Real Animal Tissues, Other customized phantoms  Other methods: Electron Density Phantoms, Rando Phantoms, IROC Phantoms, Real Animal Tissues, Other customized phantoms | Yes  No |
| 6. Other than regular target and OAR contouring, are these additional imaging techniques used to minimize patient-specific heterogeneity uncertainty (for each disease site)?  Metal artifact reduction (IMAR/OMAR)  DECT  MRI  Other (e.g. PET/CT, Proton Radiography, Prompt Gamma) | Yes  No |
| 7. What range uncertainty (in percent or absolute values) do you typically use for each disease site? | 1-10%  1-10 mm |
| 8.a Based on plan uncertainty evaluations using various overrides or robust optimization, do you ever replan or cancel treatment?  Replan  Cancel treatment | Yes  No |
| 8.b During patient specific QA, what criteria/action levels initiate the following actions?  Renormalize MU according to patient-specific QA  Replan  Cancel treatment | Gamma analysis criteria  (mm): 2 - 5 mm  Gamma analysis criteria (%): 2 - 5%  Gamma analysis criteria (threshold for ROI): 5%, 10%, 20%  Gamma analysis criteria (passing rate): 80%, 85%, 90%, 95% |
| 9.a How often do you override implant materials using the following methods?  Material Composition: Choice of CT HU threshold, Vendor provided information, Public available search information, DECT information, MRI information  Material Density: Choice of CT HU threshold, Vendor provided information, Public available search information, DECT information, MRI information  Implant Dimension: Choice of CT HU threshold, Vendor provided information, Public available search information, DECT information, MRI information | Always  Often  Sometimes  Never |
| 9.b Is there a limitation on the metal size or the uncertainty of the metal size in you clinic that prevents you from using proton treatment? | No, we do not have a hard stop on the size of metal. But we evaluate it case by case.  Yes (please comment). |
| 9.c When proton beams have to pass through the metal hardware, how do you handle the range uncertainty and dose uncertainty caused by imaging artifacts and uncertainty in estimating stopping power ratio? (You can choose multiple options that apply to your clinic).  Increase range uncertainty margin to take into account possible larger range uncertainty caused by metals (e.g. increase from 3% to 5%)  Use more beams from different passing through angles to smooth out the dose perturbations before and after the metal  Use Monte Carlo algorithm to get more accurate dose calculation  Contour the artifacts around the metal (not the metal itself) and assign the stopping power of the organ around the metal to it  Contour the metal exactly if its dimensions are known  Contour the metal approximately based on the window/level from CT and/or MRI images  Assign stopping power (from in house measurements, literature research, or calculation/estimation) to metal contour  No contour of the metal and no override of the stopping power  No consensus on the metal contour and override in our clinic. It is a clinical decision case by case based on physicists and physician's opinions. | Always  Sometimes  Never |
